# The role of the tensor veli palatini muscle in the development of cleft palate-associated middle ear problems

**DOI:** 10.1007/s00784-016-1828-x

**Published:** 2016-05-07

**Authors:** David S. P. Heidsieck, Bram J. A. Smarius, Karin P. Q. Oomen, Corstiaan C. Breugem

**Affiliations:** 1 Division of Plastic, Reconstructive and Hand Surgery, Wilhelmina Children’s Hospital, University Medical Center Utrecht, KE.04.140.0, P.O. Box 85090, 3508 AB Utrecht, The Netherlands; 2 Division of Otorhinolaryngology, Wilhelmina Children’s Hospital, University Medical Center Utrecht, Utrecht, The Netherlands

**Keywords:** Cleft palate, Eustachian tube, Otitis media with effusion, Tensor veli palatini muscle

## Abstract

**Objective:**

Otitis media with effusion is common in infants with an unrepaired cleft palate. Although its prevalence is reduced after cleft surgery, many children continue to suffer from middle ear problems during childhood. While the tensor veli palatini muscle is thought to be involved in middle ear ventilation, evidence about its exact anatomy, function, and role in cleft palate surgery is limited.

This study aimed to perform a thorough review of the literature on (1) the role of the tensor veli palatini muscle in the Eustachian tube opening and middle ear ventilation, (2) anatomical anomalies in cleft palate infants related to middle ear disease, and (3) their implications for surgical techniques used in cleft palate repair.

**Materials and methods:**

A literature search on the MEDLINE database was performed using a combination of the keywords “tensor veli palatini muscle,” “Eustachian tube,” “otitis media with effusion,” and “cleft palate.”

**Results:**

Several studies confirm the important role of the tensor veli palatini muscle in the Eustachian tube opening mechanism. Maintaining the integrity of the tensor veli palatini muscle during cleft palate surgery seems to improve long-term otological outcome. However, anatomical variations in cleft palate children may alter the effect of the tensor veli palatini muscle on the Eustachian tube’s dilatation mechanism.

**Conclusion:**

More research is warranted to clarify the role of the tensor veli palatini muscle in cleft palate-associated Eustachian tube dysfunction and development of middle ear problems.

**Clinical relevance:**

Optimized surgical management of cleft palate could potentially reduce associated middle ear problems.

## Introduction

Otitis media with effusion is very common in infants with an unrepaired cleft palate under the age of 2 years. At the time of cleft palate repair, more than 90 % of the middle ears contain mucoid material (“glue ear”) [1, 2]. Although its prevalence is reduced after surgical cleft palate repair, a significant number of children continue to suffer from middle ear disease throughout their child- and adulthood [3, 4]. Special attention is required as recurrent middle ear problems can result in tympanic membrane retraction, cholesteatoma, and (irreversible) hearing loss [3, 5, 6]. Especially during the learning phase of these infants, hearing loss can have a significant negative effect on their development of speech, language, and (social) behavior [4, 5, 7].

Optimal otological management in cleft palate patients has historically been a point of discussion, with ventilation tubes often being inserted preventively at the time of cleft palate repair. Although routine ventilation tube insertion leads to short-term hearing gain, the long-term otological outcome of these patients does not appear to be superior to outcomes of patients receiving ventilation tubes merely on indication [8, 9]. There is also evidence that a conservative approach even improves long-term otological outcome [4, 5, 8].

The high incidence of middle ear problem in cleft palate children is thought to be caused by Eustachian tube dysfunction, that is the result of aberrations of the paratubal muscles that are normally responsible for Eustachian tube opening [10–12]. The hampered Eustachian tube function results in poor ventilation of the middle ear cavity, which subsequently leads to a negative pressure and a retracted tympanic membrane with mucous secretion [13]. Eventually, this can result in otitis media with effusion, defined by the presence of middle ear effusion for at least 3 months [14]. Next to paratubal muscle abnormalities, Eustachian tube dysfunction can be caused by peritubal lymphoid hyperplasia resulting from soiling of nutrition in the nasal cavity [15]. Other potential etiologies include altered Eustachian tube compliance [16], abnormalities of the nasopharyngeal orifice [17], and other Eustachian tube tissue aberrations [18, 19].

Current surgical techniques used in cleft palate repair are primarily focused on restoring the barrier between the oral and nasal cavity and constructing a levator veli palatini muscle sling. By maximizing the levator veli palatini muscle sling function in velopharyngeal closure, the possibility of developing hypernasal speech is reduced [20]. The adjacent tensor veli palatini muscle is recognized to be involved in middle ear ventilation; however, evidence about its exact functions and anatomy remains inconclusive. Some surgeons prefer to transect the tensor veli palatini muscle (i.e., tenotomy) to assist mobilization and closure of palatal tissue during cleft repair. Others prefer to perform a tenopexy or release it by fracturing the pterygoid hamulus around which the tendon passes [21]. Controversy regarding the effect of these procedures on Eustachian tube function and middle ear ventilation however remains. A more recent study on the effects of tensor veli palatini muscle preservation on Eustachian tube function [21] prompted us to do a thorough review of the literature with regard to (1) the role of the tensor veli palatini muscle in Eustachian tube opening and middle ear ventilation, (2) anatomical anomalies in cleft palate infants related to middle ear disease, and (3) their implications for surgical techniques used in cleft palate repair.

## Methods

An electronic literature search of the MEDLINE database was conducted using a combination of the following search terms: “cleft palate and otitis media,” “cleft palate and ventilation tube,” “cleft palate and tensor veli palatini,” and “tensor veli palatini and Eustachian tube.” Our search yielded 53 relevant studies, including 6 experimental animal studies, 6 experimental studies involving humans, 8 histological studies, and 5 clinical studies that are presented in Tables 1, 2, 3, and 4. The outcomes of these studies will be discussed from here on.

**Table 1:** Overview of studies involving animal models investigating the role of the tensor veli palatine muscle in Eustachian tube opening

Authors	Year	Aim(s) of the study	Subjects	Characteristics age/weight (range)	Results	Conclusion(s)
Honjo et al. [22]	1979	Identify muscle responsible for tubal opening using EMG and electrical muscle stimulation.	12 dogs	NR/NR	Stimulation of TVP resulted in drop of pressure in middle ear space, whereas LVP stimulation did not affect pressure.	(1) TVP is the only active tubal dilator in dogs (2) LVP has no effect in ET dilation
Cantekin et al. [23]	1979	Verify results of previous studies showing the TVP as only tubal dilator.	5 juvenile macaque monkeys	NR/2.5–4 kg	(1) Stimulation of mandibular nerve produced a pressure-flow drop in ET similar as recorded during swallowing^a^. (2) Pressure-flow drops were no longer observed following TVP transection.	(1) TVP is the only active tubal dilator in rhesus monkeys. (2) Mandibular nerve innervates the TVP
Honjo et al. [24]	1980	Examining (1) synergistic action between TVP and LVP and (2) ET opening process using contrast fluid and cineradiographic analysis while stimulating the muscles.	4 macaque monkeys	NR/4–10.5 kg	(1) TVP stimulation resulted in drop of tympanal pressure, while LVP stimulation did not affect pressure. (2) LVP stimulation caused inward movement of the torus tubarius and thus widening of the pharyngeal orifice. TVP stimulation opened the proximal part of the ET through outward displacement of the lateral tubal wall.	(1) TVP is the sole ET opener. (2) LVP dilates the ET only at its pharyngeal orifice, while the TVP opens the ET by pulling the lateral wall outwards.
Cantekinet al. [25]	1980	Examining the effects of surgical TVP procedures (excision, transection, transposition) on ET function and ME status.	22 juvenile and adult macaque monkeys	NR/2–6 kg	(1) TVP excision caused chronic OME and complete tubal dysfunction. (2) TVP transection lead to abnormal ME pressure, effusion, or both with a transient, recurrent or chronic character. The ET was initially dysfunctional, however slowly regained function. (3) TVP transposition had initially similar effects as transection, though ME pathology and ET function improved more rapidly.	Surgical manipulations of the TVP created a functional ET obstruction; the severity of ET dysfunction depended on the performed surgical procedure with excision being the most harmful.
Casselbrant et al. [26]	1988	Investigate the effects of TVP paralysis (using botulinum toxin A^b^) on ET function and ME status.	8 adult macaque monkeys	NR/5–9 kg	10/12 examined ears develop flat tympanograms within 8–30 days indicating middle ear effusion (confirmed in 7 ears by tympanoscentesis). Tympanograms required 13–32 days to normalize.	Injecting botulinum toxin A into the TVP creates reversible functional ET obstruction which became evident as high-negative pressure followed by middle ear effusion.
Ghadiali et al. [27]	2003	Investigate the effects of TVP paralysis (using botulinum toxin A^b^) on ET tissue dynamics.	12 macaque monkeys	NR/2–4 kg	Loss of TVP muscle tone and stiffness resulted in significant decrease of ET opening pressure, increased ET compliance and reduced ET viscoelasticity.	Paralysis of TVP by botulinum toxin results in decreased function due to alterations of ET mechanical properties.

*ET* Eustachian tube, *EMG* electromyography, *TVP* tensor veli palatini muscle, *LVP* levator veli palatini muscle, *ME* middle ear, *OME* otitis media with effusion, *NR* not reported

^a^Swallowing was induced by pharyngeal stimulation

^b^Botulinum toxin A is known for its paralyzing effects by working on acetylcholine release at the neuromuscular junctions

**Table 2:** Overview of clinical studies involving humans investigating the role of the tensor veli palatini muscle in Eustachian tube opening

Authors	Year	Aim(s) of the study	No. of subjects	Mean age (years)	Results	Conclusions
Takahara et al. [28]	1986	Examining OME etiology in young man suffering from intracranial NHL.	1	26	(1) Tumor cells invaded lateral part of ET cartilage and resulted complete TVP destruction. LVP was free of tumor cells although pushed medially by the tumor mass. (2) Areas of various sections of the ET were measured and did not differ from normal adults.	OME most likely caused by functional ET obstruction resulting from TVP dysfunction due to tumor invasion of the muscle itself, its nerve supply or both. Mechanical ET obstruction due to tumor mass was ruled out.
Su et al. [29]	1993	Examining relationship between ET dysfunction and abnormal TVP and LVP EMG activity in patients with.nasopharyngeal carcinoma (NPC).	46	NR (range: NR)	(1) Majority of 28 symptomatic ears (tinnitus, sensation of fullness, hearing loss) showed abnormal audiological tests and tympanogram with OME^a^. (2) Abnormal TVP EMG waves were noted in 19 (68 %) of the symptomatic ears.	(1) Neurogenic TVP paralysis is associated with functional ET obstruction in NPC patients resulting in OME. (2) LVP played no significant role in active tubal opening in contrast with the TVP. (3) The TVP is the primary opener of the ET.
Sapci et al. [30]	2008	Analyzing TVP function in patients with chronic middle ear pathology^b^ versus a control group using EMG.	42	44. (range: NR)	(1) TVP EMG in affected ears did not significantly differ from healthy ears. (2) In the only patient with palate pathology^c^, TVP EMG activity on both sides were significantly lower compared with the mean values of the control group.	(1) TVP activity is normal in patients with chronic unilateral middle ear. (2) In patients with palate pathology, dysfunction of the TVP might play a role in the development of chronic ME pathology.
Chang et al. [31]	2013	Functional evaluation of LVP and TVP in patients with chronic unilateral tubal dysfunction.	10	39 (range 17–58)	(1) TVP EMG activity on the affected side was normal in 9/10 patients during phonation or swallowing. (2) In 5/10 patients LVP EMG activity on the affected side was decreased during phonation or swallowing.	Reduced activity of the LVP may be related to ET dysfunction in patients with chronic unilateral tubal dysfunction.
Hanzel et al. [32]	2012	Pilot-study assessing the use of sonotubometry and nasopharyngeal endoscopy to investigate ET opening in healthy adults.	1/17	NR (range 23–52)	One patient with significant control over LVP and TVP contraction received further assessment. (1) LVP contraction showed to elevate the palate and medially rotated the posterior cushion, dilating the posteromedial wall of the ET, but not causing ET opening. (2) TVP contraction resulted in ET opening.	(1) TVP activity is required for ET opening. (2) LVP muscle movement in the soft palate affected the posteromedial wall of the ET without causing actual tubal opening.
Alper et al. [33]	2012	Determining the role of the TVP and LVP in ET opening using sonotubometry, nasopharyngeal endoscopy and EMG in healthy adults.	15	36 (range 19–54)	(1) TVP EMG activity was higher but had a shorter duration compared to LVP EMG activity during swallowing. (2) First anatomical change observed during a swallow was elevation of soft palate during onset of LVP contraction. (3) Posterior displacement and axial rotation of the medial lamina of the ET cartilage mirrored LVP activity with a slight delay to onset of a swallow. (4) The posterior displacement of the lateral wall of the ET lumen corresponded to TVP contraction and maximum ET opening.	(1) TVP activity is associated with peak ET opening. (2) LVP activity occurs before TVP activity and peak ET opening. (2) LVP contractions are associated with movement of the soft palate, anterior ET orifice and rotation of the ET cartilage.

*EMG* electromyography, *ET* Eustachian tube, *LVP* levator veli palatini muscle, *OME* otitis media with effusion, *NHL* non-Hodgkin lymphoma, *TVP* tensor veli palatini muscle, *NR* not reported

^a^OME was identified by performing myringotomy

^b^Chronic middle ear pathology included chronic otitis media, retraction of tympanic membrane, OME, and atelectasis

^c^Patient in this suffered from a bifid uvula and submucosal palate burn

**Table 3:** Anatomical abnormalities of the tensor veli palatini muscle and Eustachian tube in cleft palate patients

Anatomical abnormalities in CP patients	Study	Year	No. of CP specimens	Age of CP patients	No. of controls	Age of controls
Smaller TVP to ET cartilage insertion ratio	Matsune et al. [12]	1991	10	32 gest. weeks–3 years	20	33 gest. weeks–2 years
Smaller ET cartilage area ratio: LL/ML	Matsune et al. [19]	1991	10	32 gest. weeks–3 years	20	33 gest. weeks–2 years
ET tubal lumen is less curved	Matsune et al. [19]	1991	10	32 gest. weeks–3 years	20	33 gest. weeks–2 years
	Shibahara & Sando [11]	1988	8	24 gest. weeks–6 weeks	8	Age-matched
Greater cartilage cell density^a^	Yamaguchi et al. [34]	1990	11	24 gest. weeks–3 years	24	24 gest. weeks–3.5 years
Less elastin at the “hinge portion” of ET cartilage	Matsune et al. [19]	1992	6	3 days–3 years	13	6 aged 19–52 years, 7 aged 2 days–2 years
Smaller ET cartilage LL and ML volume and volume ratio (LL/ML)	Takasaki et al. [35]	2000	9	1 day–1 month^b^	16	Up to 1 month^b^
Smaller angle at which the TVP pulls the ET lumen	Shibahara & Sando [11]	1988	8	24 gest. weeks–6 weeks	8	Age-matched
Reduced sensitivity of ET to TVP forces and increased sensitivity to periluminal mucosal tissue stiffness	Sheer et al. [36]	2012	5	1 month–2.5 years	4 6	4–10 years 19–76 years
(1) Increased nasopharynx space (2) Smaller hamulus to lateral pterygoid plate width. (3) Differences in angulation hamulus^a^.	Rajion et al. [37]	2012	29	0–12 months	12	Age-matched

*CP* cleft palate, *ET* Eustachian tube, *gest.* gestational, *LL* lateral lamina, *ML* medial lamina, *TVP* tensor veli palatini muscle

^a^Not statistically significant

^b^Takasaki et al. did not mention gestational age at which the children deceased

**Table 4:** Clinical studies reporting possible implications for surgical techniques used in cleft palate repair in relation to otological outcome

Authors	Year	Aim of the study	Study type	No. of subjects	Mean age	Results	Conclusions
Sehhati-Chafai-Leuwer et al. [3]	2006	Evaluating otological status and TVP integrity in adult patients with repaired cleft palate usingotomicroscopy and MRI.	Case-control study	*N* = 15 (1 subject with unrepaired cleft)	25 years (range 13 – 45)	(1) All patients with repaired cleft and chronic ME pathology (*n* = 7) showed non-intact TVP (neither towards the LVP nor towards the hamulus) on MRI. Hamulus was undetectable on MRI in 4/7 cases. (2) All patients with repaired cleft palate and normal otomicroscopy findings (*n* = 7) had intact TVP and hamulus on MRI. (3) The patient with unrepaired cleft showed only mild ear pathology with a complete TVP.	Evident correlationbetween post-treatmentintegrity of the TVP and ME status, with all patients having an incomplete TVP suffering from middle ear pathology.
Flores et al. [21]	2010	Comparing effects of TVP preservation^a^, transection^b^, and “tensor tenopexy”^c^ on ET function.	Case-control study	*N* = 147 TVP preservation (*n* = 64) vs. TVP transection (*n* = 31) vs. tensor tenopexy (*n* = 52)	Subjects followed from age 1–7 years	(1) Decreased need ventilation tube insertion from age >4 years in the tenopexy group compared with the transection group. (2) Decreased need for ventilation tube insertion in the tenopexy group compared with the tensor preservation group (significant at age 4-5 years). (3) Decreased need for ventilation tube insertion in the tensor preservation group compared with the transection group (significant at age 6-7 years).	(1) Tensor preservation and tensor tenopexy significantly improve ME status compared to tensor transection. (2) Tension tenopexy seems to have the most beneficial effects on ME status.
Tiwari et al. [38]	2013	Evaluating the effects of tensor tenopexy on ET function and preventing hearing loss.	Randomized controlled trial	*N* = 17 tensor tenotomy (*n* = 8) vs. tensor tenotomy with tenopexy (*n* = 9)	NR (range: 9–24 months)	No significant difference in hearing loss and middle ear effusion between both groups at follow-up of 3, 6, 9 and 12 months.	Tenopexy was not found to be helpful in maintaining ET function or preventing hearing loss under the age of 12 months.
Bütow et al. [39]	1991	Evaluating the effects of TVP tension sling^d^ on ME status.	Case-control study	*N* = 39 tension sling (*n* = 19) vs. controls without sling (*n* = 20)	NR (range 6 – 36 months)	(1) ME status of the controls after surgery is not significant improved compared to ME status of the cases prior surgery. (2) ME status of the tension sling cases significantly improved at 9 and 18 months after surgery compared to the control group.	TVP tension sling seems to have beneficial effects on ME status.
Kane et al. [40]	2000	Examining the effect of hamulus fracture on outcome of palatoplasty in cleft palate patients	Randomized controlled trial	*N* = 161 hamulus fracture (*n* = 85) vs. no hamulus fracture (*n* = 76)	25 months (range: NR)	No significant difference in oral mucosa dehiscence rate and fistula occurrence between both groups.	Hamular fracturing during palatoplasty does not affect the occurrence of complications.

*MRI* magnetic resonance imaging, *TVP* tensor veli palatini muscle, *ME* middle ear, *ET* Eustachian tube

^a^TVP preservation—cleft palate repair with construction of the levator sling (no damage to the TVP or its tendon)

^b^Tensor transection—TVP tendon transection and levator sling construction

^c^Tensor tenopexy—surgical technique involving isolation of the TVP, medially displacement of the TVP tendon, suturing the tendon under tension to the hamulus, transecting the tendon medially from the hamulus, and construction of the levator sling

^d^TVP tension sling—procedure during cleft palate surgery during which a suture sling is inserted around the tendon of the TVP medial to the hamulus at one side, then wrapping it around the tendon of the TVP at the other side followed by tying the ends together under maximal tension

## Evaluation

Before the most relevant studies retrieved from our search will be discussed, a short overview of the related anatomy and opening mechanism of the Eustachian tube is given.

### Eustachian tube and tensor veli palatini muscle anatomy

**Fig. 1:**
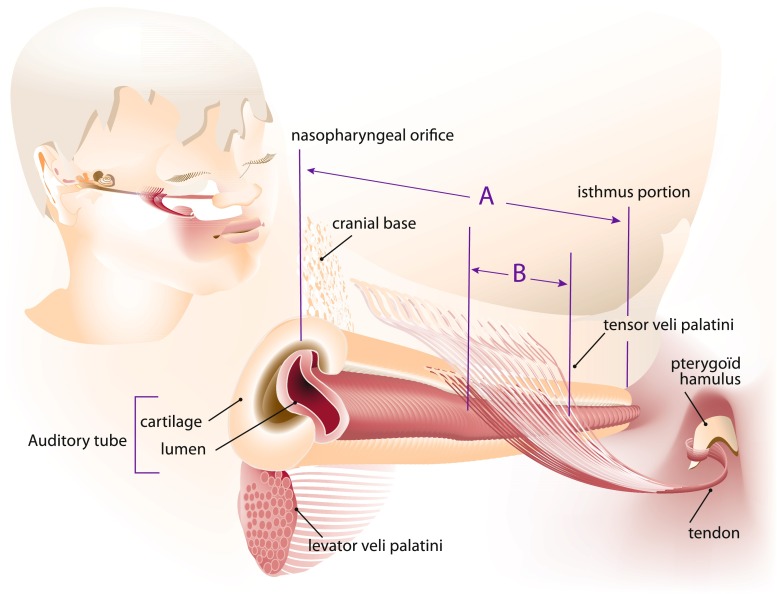
The tensor veli palatini muscle originates from the cranial base and lateral side of the auditory tube (Eustachian tube). In this figure, the tensor veli palatini muscle’s auditory tube origin is represented at the cartilaginous and membranous parts of the lateral auditory tube. Before entering the soft palatum, the tensor veli palatini muscle tendon wraps itself around pterygoid hamulus. *Insertion ratio*: The insertion ratio as described by Matsune and Sando [13] is calculated by the length of Eustachian tube cartilage involved by insertion of the tensor veli palatini muscle at the auditory tube (*line B*) divided by the total length of the Eustachian tube (*line A*); the length of the auditory tube cartilage from the nasopharyngeal end to isthmus portion. (*Figure adapted from Matsune* et al. [13])

**Fig. 2:**
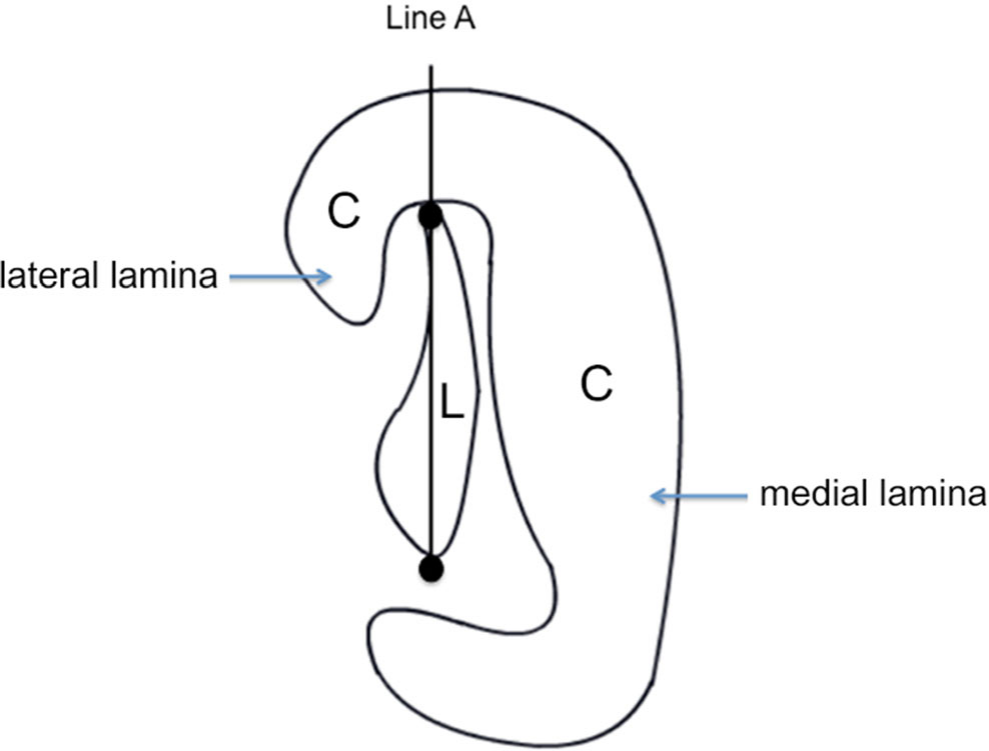
*C* Eustachian tube cartilage, *L* Eustachian tube lumen, *Line A* connection between two most distant points of Eustachian tube lumen; *Line A* separates the Eustachian tube cartilage into the lateral lamina (LL) and the medial lamina (ML); LL/ML ratio: Area of lateral lamina divided by the area of the medial lamina. (*Adapted from Matsune* et al. [19])

**Fig. 3:**
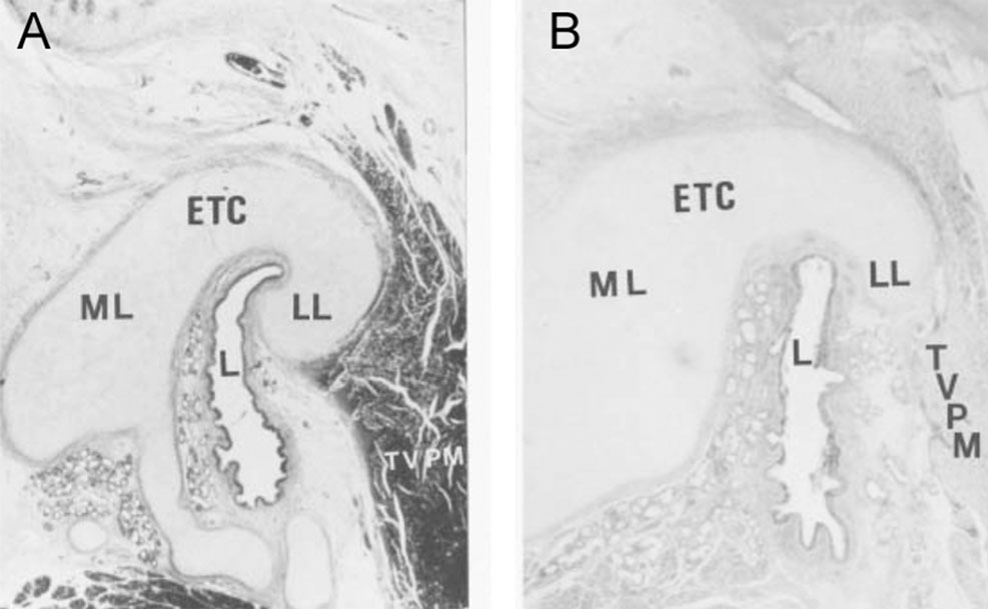
Photomicrograph of cross sections through the midcartilaginous portion of the Eustachian tube: **a** control case (6-week old female) and **b** cleft palate case (7-week old male). The photomicrographs show differences in curvature of the lumen and cross-sectional area of the Eustachian tube development of the cartilage between the normal child and the child with a cleft palate (hematoxylin-eosin stain). *ETC* Eustachian tube cartilage, *L* Eustachian tube lumen, *LL* lateral lamina of Eustachian tube cartilage, *ML* medial lamina, *TVPM* tensor veli palatini muscle. (Reproduced with permission from Matsune. [19])

The course of the Eustachian tube—from the middle ear towards nasopharynx—is anterior, inferior, and medial (Fig. 1). The tube opens in the nasopharynx just posterior to the inferior turbinate. Interior of the tube is a mucosal lining surrounded by cartilage, with the most lateral third of the tube being surrounded by a bony exoskeleton [17]. A heavy sheath of soft tissue overlies the cartilage [41] and paratubal muscles (i.e., tensor veli palatini, levator veli palatini, salpingopharyngeus muscle, tensor tympani muscle, and medial pterygoid muscle) [42, 43]. The cartilage is C-shaped in cross-section with its concave side lateral, inferior, and anterior and can be divided in a medial and lateral part called the medial and lateral lamina (Figs. 2 and 3a) [19]. Only the superior part of the lateral lumen is surrounded by cartilage while the larger inferior membranous part is not [12].

The tensor veli palatini muscle originates from the cranial base and the lateral side of the auditory tube [43–45] (Fig. 1). These two origins give rise to two muscle layers which are easily separable due to the parallel course of their muscle fibers [44]. The cranial base origin extends from the scaphoid fossa of the great wing of the sphenoid bone anteriorly to the sphenoid spine posteriorly. Different opinions exist about the tensor veli palatini muscle’s auditory tube origin. Some researchers reported an attachment to both the cartilaginous and membranous parts of the lateral Eustachian tube [15, 41, 45, 46], while others concluded that only the cartilaginous part is the true origin of the tensor veli palatini muscle [12, 44, 47]. Furthermore, Huang et al. [45] found that the tensor veli palatini muscle is attached to both parts of the lateral Eustachian tube over its entire length, while Ishijima et al. [48] concluded that the anterior part of the tensor veli palatini muscle has no attachment to the lateral lamina. Abe et al. [44] even described five different types of tensor veli palatini muscle origins. Barsoumian et al. [49] considered the dilatator tubae muscle, connecting the tensor veli palatini muscle to the lateral wall of the Eustachian tube, as a distinct structure from the tensor veli palatini muscle despite connective tissue alliance and intermingling of some muscle fibers. In a larger study, Abe et al. on the other hand found no evidence for distinction between the dilatator tubae muscle and the tensor veli palatini muscle [44]. Fibers of the two tensor veli palatini muscle layers gradually become one tendon wrapping itself around the pterygoid hamulus in an almost 90° turn before entering the soft palate (Fig. 1). The tendinous fibers fan out to form the horizontal palatine aponeurosis which inserts to the dorsal edge of the hard palate. The maxillary tuberosity (behind the second molar) and pharyngeal mucosa (behind the pharyngeal arch) are also points of insertion [44]. In anatomical studies, Abe et al. [44] found no anchoring of the tendon to the hamulus, whereas Huang et al. [45] did find some insertion into the hamulus. Flores et al. [21] provided clinical evidence that the tensor veli palatini muscle exists as a partial pulley around the hamulus.

### Opening mechanism of the Eustachian tube

The Eustachian tube is closed at rest as a result of the elastic connective tissue compliance in the tubal walls, extramural pressure of adjacent structures, and the tonus of the paratubal musculature [50, 51]. The underlying mechanism responsible for tubal opening, which takes place during yawning and swallowing, still requires clarification. Although in earlier literature tubal dilation was solely ascribed to tensor veli palatini muscle contraction [22, 52], most researchers consider it to be a result from a synergistic action between the tensor veli palatini and levator veli palatini muscle [17, 24, 43, 45, 46, 53]. The tensor veli palatini muscle action is assumed to be an isotonic contraction resulting in a direct increase of the lumen and a downwards traction on the lateral cartilage producing a rotational force on the medial cartilage [45]. Several studies suggest that the levator veli palatini muscle participates in opening of the most anterior Eustachian tube portion at the pharyngeal orifice [17, 43, 45, 47, 48, 50].

### Role of the tensor veli palatini muscle in middle ear ventilation

In the recent literature, there seems to be a general consensus that the tensor veli palatini muscle is the primary opener of the Eustachian tube and thereby has a key role in middle ear ventilation. The first purpose of this literature review was to investigate the tensor veli palatini muscle’s role in Eustachian tube opening and middle ear ventilation. Tables 1 and 2 present an overview of relevant experimental studies involving animals and human subjects assessing the exact role of the tensor veli palatini muscle in tubal opening.

The first experimental study investigating the muscles responsible for Eustachian tube function was conducted by Honjo et al. [22]. The authors observed that minor electrical stimulation of the tensor veli palatini muscle in dogs consistently produced tubal opening which was registered by a sudden drop in air pressure in the middle ear space. For these measurements, the eardrums were perforated followed by closure of the external ear canal and insertion of a small tube conducting the ear pressure to a pressure transducer. Similar results were found in comparable experiments on macaque monkeys [24]. Moreover, Cantekin et al. [23] observed that stimulating the mandibular nerve in rhesus monkeys resulted in tubal opening whereas nerve stimulation after complete transection of the tensor veli palatini muscle no longer had an effect.

To examine a possible synergistic action of the tensor veli palatini muscle and levator veli palatini muscle and to visualize the opening process of the tube, Honjo et al. [24] injected contrast fluid through perforated tympanic membranes in macaque monkeys. The muscles were independently stimulated while filming the tube at 24 frames/s. Cineradiographic analysis showed a consistent opening pattern. The levator veli palatini muscle did not change the tubal lumen but did cause dilation of the nasopharyngeal orifice. Contraction of the tensor veli palatini muscle caused an outward displacement of the lateral tubal wall by which the Eustachian tube was opened.

The significance of a well-functioning tensor veli palatini muscle is underlined in three studies in which a functional Eustachian tube obstruction model was created by affecting the muscle through surgical manipulation or injection of paralyzing agents [25–27]. Cantekin et al. [25] performed various surgical alterations of the tensor veli palatini muscle in rhesus monkeys: (1) complete excision of the muscle, (2) transection of the superficial muscle bundle, and (3) transposition, realized by isolating the tendinous portion of the tensor veli palatini muscle and lifting it over the hamulus, giving it a more medial position. In the large majority of the ears in the excision group, middle ear effusion developed within 5 weeks and in most cases became a chronic condition. Eustachian tube function tests revealed a complete failure of tubal dilation upon swallowing. Tensor veli palatini muscle transection of the superficial muscle bundle resulted in development of abnormal middle ear pressure, otitis media with effusion, or both. However, in only 3 of the 14 ears, otitis media with effusion became a chronic or recurrent problem. During a period of 20 weeks after surgery, the impaired tubal opening was followed by gradual improvement indicating tensor veli palatini muscle healing. Tensor veli palatini muscle transposition resulted in similar outcomes as transection; however, improvement of tubal function was seen much sooner. Casselbrant et al. [26] used botulinum toxin injections to paralyze the tensor veli palatini muscle in rhesus monkeys. This caused a reversible functional Eustachian tube obstruction which became evident as a high negative pressure followed middle ear effusion. Ghadiali et al. [27] also used botulinum toxin in experiments on macaque monkeys and observed that paralyzing the tensor veli palatini muscle resulted in changed Eustachian tube tissue mechanics. Loss of muscle tone and stiffness caused decreased Eustachian tube opening pressure, increased Eustachian tube compliance, and decreased Eustachian tube viscoelasticity, together contributing to diminished Eustachian tube opening function.

All the abovementioned experimental animal studies [22–27] conclude that the tensor veli palatini muscle plays an essential role in Eustachian tube opening and thus middle ear ventilation. Despite the established similarity of tubal anatomy in primate extrapolation of results from primate models towards humans remains controversial [46]. Clinical studies addressing the role of the tensor veli palatini muscle on Eustachian tube function in humans are presented in Table 2. Takahara et al. [28] and Su et al. [29] reported cases of patients with non-Hodgkin lymphoma and nasopharyngeal carcinoma showing destruction of the tensor veli palatini muscle or its innervating nerves caused by invading tumor cells. Both authors observed changes resulting in functional Eustachian tube obstruction followed by otitis media with effusion in the majority of patients [28, 29]. Sapci et al. [30] evaluated the tensor veli palatini muscle function in patients with chronic middle ear disease compared to healthy individuals using electromyography (EMG). No significant difference in tensor veli palatini muscle EMG activity was found between affected and healthy ears. However, one case showed a significantly decreased EMG activity compared to the control group. This happened to be a patient with palate pathology (bifid uvula and submucosal palate burn), with the authors suggesting muscular dysfunction of the tensor veli palatini muscle being a causal factor. Chang et al. [31] performed a similar study evaluating the tensor veli palatini and levator veli palatini muscle function by measuring EMG activity in patients with chronic unilateral tubal dysfunction. In the large majority of patients, no signs of decreased tensor veli palatini muscle activity were found on the affected side. The levator veli palatini muscle activity however was decreased in half of the patients, suggesting that reduced activity of the levator veli palatini muscle may play a role in Eustachian tube dysfunction in patients with chronic unilateral tubal dysfunction. In a study using a combination of sonotubometry and nasopharyngeal endoscopy, Handzel et al. [32] investigated Eustachian tube opening in healthy adults during swallowing, yawning, and phonation. One patient with significant control over contractions of the levator veli palatini muscle and tensor veli palatini muscle received further examination. Contracting the levator veli palatini muscle showed to elevate the palate and medially rotate the posterior cushion, dilating the posteriomedial wall of the Eustachian tube however not causing actual tubal opening. Tensor veli palatini muscle contraction on the other hand did result in Eustachian tube opening. These findings were confirmed by Alper et al. [33] who investigated a similar study group however using a combination of EMG, sonotubometry, and endoscopy. The authors concluded that tensor veli palatini muscle contraction was related to peak Eustachian tube opening, whereas levator veli palatini muscle activity preceded tensor veli palatini muscle activity and thus actual Eustachian tube opening. Levator veli palatini muscle contraction showed to be associated with movement of the soft palatum and anterior Eustachian tube orifice and rotation of the Eustachian tube cartilage. This however did not result in actual Eustachian tube opening.

The abovementioned studies [28–33] provide solid evidence confirming the essential role of the tensor veli palatini muscle in Eustachian tube opening in animal models and human adults without a cleft palate. Based on conclusions from both animal and human experimental studies, we conclude that impairment of the tensor veli palatini muscle leads to dysfunction of Eustachian tube opening, impaired middle ear ventilation, and most likely otitis media with effusion. The studies of Alper et al. [33] and Handzel et al. [32] also indicate involvement of the levator veli palatini muscle in Eustachian tube opening although most probably at a different stage that precedes the actual opening.

### Role of anatomical anomalies on Eustachian tube dysfunction in cleft palate patients

Our second purpose was to examine to what extent anomalies in the tensor veli palatini muscle, Eustachian tube, or surrounding structures contribute to the development of Eustachian tube dysfunction and middle ear problems in cleft palate patients. In the early 1960s, several factors were proposed to play a causal role in Eustachian tube dysfunction and frequent middle ear pathology in cleft palate patients, including poor tensor veli palatini muscle development and the absence of a firm attachment of the tensor veli palatini muscle to the Eustachian tube [51]. In later studies, a functional Eustachian tube obstruction was still considered the most likely cause of otitis media with effusion in cleft palate patients and was suggested to be the result of an abnormal Eustachian tube opening mechanism, increased tubal compliance, or both [26, 28]. Using a radiopaque contrast medium in cleft palate patients, it was demonstrated that upon swallowing, the Eustachian tube failed to open at its nasopharyngeal orifice [50]. Additionally, nasopharyngoscopy showed abnormalities of the nasopharyngeal orifice, namely a slightly different location, smaller size, and a high frequency of hypoplasia of the torus tubarius (the cartilaginous portion of the orifice). Moreover, the tubal cartilage in cleft palate patients showed no movement during swallowing, whereas normally, an upward movement is expected that results in an increased Eustachian tube lumen patency [17].

In Table 3, results are summarized from histological studies that investigated anatomical abnormalities of the tensor veli palatini muscle and Eustachian tube in cleft palate patients. Matsune et al. [12, 18, 19] conducted several studies to investigate a possible relationship between anomalies of the Eustachian tube or related structures and the frequent occurrence of otitis media with effusion in cleft palate patients. First, the authors investigated to what extent the tensor veli palatini muscle inserts into the tubal cartilage of cleft palate children and how this differed from normal children. Insertion was defined as “incorporation of the tendon of the tensor veli palatini muscle into the perichondrium of the tip of the lateral lamina (LL) of the tubal cartilage” [12]. The tip of the lateral lamina is the most inferior part when cross-sectioned due to the C-shape of the tubal cartilage. The length of the insertion (along the long axis of the Eustachian tube cartilage) was calculated for each specimen by determining the insertion in a sequence of histological sections. Then, the insertion ratio was calculated as illustrated by Fig. 1. A significant difference in tensor veli palatini muscle insertion was observed in cleft palate patients compared to the control group. Furthermore, all insertions in cleft palate patients consisted of fewer muscle and tendon fibers. The average insertion ratio in cleft palate patients (excluding four cases without insertion) was significantly smaller compared to the control group [12]. These particular cases showed to have more severe middle ear and Eustachian tube inflammation. As a result of ineffective transmission of muscle force to the cartilage, we expected a worse middle ear status and more Eustachian tube inflammation in cleft palate patients without insertion compared to patients with (poor) tensor veli palatini muscle insertion. This however was not confirmed by the results of this study [12]. As was appropriately remarked by the authors, a larger number of specimens must be used in order to confirm their findings. The authors did not mention any involvement of the upper lip in the cleft palate patients. It was also unclear whether the cleft palate patients were isolated defects or part of a broader defect or syndromal genetic disorder. In our opinion, this is essential information as in cases of a syndromal disorder, the anatomical deviation in the palate region tends to be more extensive [54].

In another study, Matsune et al. [19] investigated the lateral lamina development of the Eustachian tube’s tubal cartilage in cleft palate patients. Various measurements were obtained in the cross-sectioned mid-cartilaginous portion of each specimen resulting in calculation of a lateral lamina/medial lamina ratio as shown in Fig. 2. The mean ratio in cleft palate patients was significantly smaller compared to the control group [19]. These results however are inconsistent with findings of Shibahara and Sando [11], reporting no significant difference when comparing the cross-sectional area of lateral lamina in the specimens of cleft palate patients with age-matched controls. Takasaki et al. [35] though mentioned the difficulty to cut histological sections consistently in a plane perpendicular to the axis of the Eustachian tube and therefore measured the cross-sectional volume of the Eustachian tube cartilage instead of the area. In this study, the lateral lamina volume and volume ratio (lateral lamina/medial lamina) were significantly smaller in cleft palate patients compared to the control group. This supports the hypothesis proposed by Matsune et al. [18] that a poor lateral lamina development might be an important factor in tubal dysfunction in cleft palate patients.

Additionally, Matsune et al. [19] found that the tubal lumen shape was significantly less curved in cleft palate patients compared to controls (Fig. 3). The patients that did show a curved lumen had a significantly smaller luminal curve ratio. These results however are highly dependent on the plane in which the histological sections are cut. As earlier mentioned, consistently making cuts in the plane perpendicular to the long axis of the Eustachian tube for all sections however is challenging.

Shibahara and Sando [11] also performed measurements in histological sections of the Eustachian tube and surrounding tissues and found several anatomical differences in cleft palate patients: (1) a significant smaller angle between the axis of the tensor veli palatini muscle and superior portion of the Eustachian tube lumen, (2) a significant wider angle between the axis of the lateral lamina and medial lamina, (3) a significant smaller angle between the axis of the tensor veli palatini muscle and lateral lamina, and (4) a significant wider angle between the axis of the superior and inferior parts of the Eustachian tube lumen [11]. The last findings are in accordance with results of Matsune et al. [19] demonstrating that the tubal lumen is less C-shaped in cleft palate patients. The angle at which the tensor veli palatini muscle inserts to the cartilage in cleft palate patients is narrower than normal and thereby leading to a less efficient pull force resulting in Eustachian tube dysfunction.

Although cartilage cell density in cleft palate individuals is reported to be higher than normal [55], a study from Yamaguchi et al. found no significant differences between cleft palate patients and healthy age-matched controls [34]. The Eustachian tube cartilage has an area where the tissue is rich in elastin, located on the luminal side at the intermediate between the lateral lamina and medial lamina (the “hinge portion”) [18]. This supports the hypothesis that passive closure of the tube is due to elasticity of the cartilage at this hinge portion [51], and that returning of the tubal lumen to its neutral position before the following tensor veli palatini muscle contraction might be essential for an efficient opening and thus for normal ventilation [18]. Matsune et al. investigated differences in elastin distribution by examining series of histology sections (cut perpendicular to the long axis of the Eustachian tube) from specimens obtained from healthy children and adults and children with a cleft palate. Staining for elastin showed a significantly greater density at the hinge portion in adults compared to children, and a significantly greater density in non-cleft children compared to cleft palate children [18]. The lesser amount of elastin in children and even less so in cleft palate patients might be an important factor in causing otitis media with effusion through lacking the Eustachian tube getting in its neutral position before the next contraction of the tensor veli palatini muscle. In that way, an inefficient transmission of force would lead to insufficient tubal dilation.

Sheer et al. [36] used computational models based on human histologic specimens and three-dimensional reconstructive techniques to study the Eustachian tube function in cleft patients, healthy children, and adults. The authors concluded that in young children with cleft anomalies, the Eustachian tube opening is less sensitive to tensor veli palatini muscle forces when compared to adults, while remaining sensitive to changes in periluminal mucosal tissue stiffness. Their finding implies that changes in cartilage and periluminal mucosal tissue might be of greater importance in proper Eustachian tube function in these groups.

Based on the earlier mentioned studies, we can conclude that anatomical abnormalities of the tensor veli palatini muscle and Eustachian tube are considered to play a causal role in functional Eustachian tube obstruction in cleft palate patients. Morphological variations in the surrounding structures however may also play a causal role in Eustachian tube dysfunction. Rajion et al. [37] performed a study assessing three-dimensional CT images of the nasopharyngeal area in unoperated cleft lip and palate patients and found anatomical variations that may compromise the dilatory mechanism of the Eustachian tube. This included an increased width of the nasopharynx which may lead to compression of the surrounding structures including the Eustachian tube. Also, variations of the pterygoid plate and hamulus were found, potentially leading to alteration of the tensor veli palatini muscle’s origin and orientation. To what extent each factor contributes to Eustachian tube dysfunction and otitis media with effusion in cleft palate patients remains unclear.

### Implications for cleft palate surgery

The final goal of this study was to review literature regarding the implications of surgical techniques used in cleft palate repair on the development of post-treatment middle ear pathology. Table 4 provides an overview of five clinical studies with results regarding possible implications for cleft palate surgeons.

Sehhati-Chafai-Leuwer et al. [3] used MRI imaging to study the tensor veli palatini muscle in cleft palate patients who had undergone palatal surgery. MRI images in patients suffering from chronic middle ear pathology showed an incomplete tensor veli palatini muscle that lacked continuity towards the levator veli palatini muscle and hamulus. Contrary to this, an intact tensor veli palatini muscle was seen in patients without evident ear pathology [3]. Results from this imaging study empower the correlation between post-surgical integrity of the tensor veli palatini muscle and reduced incidence of middle ear pathology. However, as data concerning the used surgical cleft repair techniques were not reported, the results of this study are more difficult to interpret.

Several surgical techniques have been proposed in an attempt to improve post-treatment tubal function in cleft palate patients in addition to the commonly performed cleft palate repair with levator sling construction. The tensor tenopexy technique consists of (1) identification of the tensor veli palatini muscle, (2) dissection from its aberrant attachment to the posterior edge of the hard palate, and (3) medial displacement of the tensor veli palatini tendon and suturing it under tension to the hamulus. After tensor tenopexy, the tendon is cut medial to the hamulus and the levator sling construction is performed. Medial displacement of the tensor veli palatini muscle tendon theoretically results in a more open configuration (flexed position) of the Eustachian tube lumen and improved middle ear ventilation. Some surgeons consider tensor tenopexy in cleft repair unwarranted as the tensor veli palatini muscle tendon has fibrous attachments to the hamulus and tensor transection will therefore not have negative effect on Eustachian tube opening [49]. In spite of this, Flores et al. demonstrated that the tensor tenopexy technique, when compared with levator sling reconstruction and tensor transection alone, decreases the need for post-treatment ventilation tube insertion. This became significant at the age of 4 years and even increased until the age of 7 when follow-up ended [21]. A similar trend was seen when comparing the tensor tenopexy group with a group that underwent levator sling reconstruction with tendon preservation, showing significant differences at ages 4 and 5 years. As was appropriately remarked by the authors, this study was limited by its small multicenter study character and the lack of speech capacity testing in the tensor tenopexy group.

Tiwari et al. [38] also investigated the effect of tensor tenopexy compared to tensor tenotomy, however reported no significant difference in hearing loss and middle ear effusion between both groups at a follow-up of 3, 6, 9, and 12 months. The results from this study implicate that the possible benefits of tensor tenopexy on middle ear disease might only become notable from a more advanced age (4+ years).

Beneficial effects from exerting an immediate tension on the tensor veli palatini muscle were reported by Bütow et al. [39] This was done by placing a suture sling around the tendon of the tensor veli palatini muscle medial to the hamulus at one side, wrapping the sling around the tendon of the tensor veli palatini muscle at the other side followed by tying the ends together under maximal tension. Cleft palate patients showed a significantly improved middle ear status at 9 and 18 months post-surgery compared to the controls [39].

Dissection or dislocation of the tensor veli palatini muscle is often performed to reduce the incidence of post-operative fistula formation and oral mucosa dehiscence or to make the dissection of the levator muscle easier. However, no studies have provided empirical proof of the positive effects of this technique. Kane et al. examined the effects of hamular fracturing on the outcome of palatoplasty and found no differences in oral mucosa dehiscence rate or fistula occurrence rate. Although the authors also found no significant differences in speech results or otological outcomes, follow-ups were only performed up to the age of 3 years [40].

Based on the present literature, preservation of the tensor veli palatini muscle integrity during cleft palate repair seems to improve the long-term otologic outcome of cleft palate patients [3, 45, 56]. However, there is a lack of evidence of the positive effects of tensor preservation in children younger than 4 years. Sheer et al. [36] put forward that cleft palate children seem to have altered Eustachian tube tissue dynamics and that augmenting tensor veli palatini muscle forces may not be an effective treatment strategy to treat Eustachian tube dysfunction. The same applies for augmenting levator veli palatini muscle forces. According to the authors, surgical or pharmaceutical techniques that are able to reduce periluminal mucosa tissue stiffness could hypothetically be effective in improving Eustachian tube function in these patient groups. Future research should evaluate the effectiveness of such interventions in cleft palate patients. Similar interventions have been reported in the literature for related pathology, i.e., the use of laser ablation to treat hypertrophic mucosa in adult patients with persistent Eustachian tube dysfunction [57]. Moreover, treatment with enzyme injections used to degrade components of the extracellular matrix has been reported to alter the elasticity of soft tissue structures [58]. Also, the prevention of nasal soiling with food could potentially decrease Eustachian tube dysfunction as it can lead to peritubal hyperplasia and mucosal stiffness [15]. Future clinical studies evaluating effects of tensor veli palatini muscle preservation in cleft palate repair on middle ear pathology should follow patients at least for 4–7 years, as the positive effects of tensor veli palatini muscle preservation have shown to become evident until after several years post-treatment.

Most studies assessing the role of the tensor veli palatini muscle in Eustachian tube opening are performed on healthy patients and might not be extrapolated to cleft palate patients due to variations in anatomy. More studies are mandatory to elucidate the anatomical abnormalities found in cleft palate patients and how these influence the function of the tensor veli palatini muscle and Eustachian tube.

## Conclusion

Studies discussed in this review underline the important role of the tensor veli palatini muscle in opening of the Eustachian tube. The correlation between tensor veli palatini muscle integrity and long-term middle ear status makes it advisable not to release the tendon from the hamulus during cleft palate repair. Anatomical variations in cleft palate children may alter the effect of tensor veli palatini muscle on the dilatory mechanisms of the Eustachian tube and might contribute to recurrent otitis media with effusion as well. This review demonstrates that more research is warranted to clarify the role of the tensor veli palatini muscle within the cleft palate population in relation to the development of middle ear disease.
